# Multilocus analysis of GAW15 NARAC chromosome 18 case-control data

**DOI:** 10.1186/1753-6561-1-s1-s11

**Published:** 2007-12-18

**Authors:** Sharon R Browning, Jessica Thomas

**Affiliations:** 1 Department of Statistics, The University of Auckland, Private Bag 92019, Auckland, New Zealand

## Abstract

The Genetic Analysis Workshop 15 rheumatoid arthritis data included a set of 460 cases and 460 controls genotyped at 2300 closely spaced markers on a 10 megabase region of chromosome 18q. We conducted a multilocus analysis of these data using a localized haplotype clustering method that adapts to linkage disequilibrium structure and can be applied to large, densely genotyped data sets such as this one. We found a protective haplotype carried by 33 individuals that was significantly associated with rheumatoid arthritis in these data after adjusting for multiple testing. This haplotype was located less than 500 base pairs upstream of the *CCBE1 *gene. The association was not detected using single-marker tests, but could be found using a variety of multilocus tests.

## Background

Multilocus data analysis has potential for improved power over single marker testing for association studies. Relative power of single-marker and multilocus methods is determined by a number of factors [1], particularly the frequency of the causal variant, which is a proxy for the age of the mutation [2]. Recent work has demonstrated that haplotypic methods can have higher power than single-marker methods for ungenotyped causal variants with population frequencies of 5% or less, under realistic scenarios [3]. In the absence of information about the frequency of a causal variant, a good strategy is to apply both single-marker and haplotypic cluster tests.

Multiple testing adjustment is critical for analyses involving large numbers of tests, in order to determine whether the most significant associations are likely to be true associations or are only noise. For highly correlated tests a Bonferroni correction is excessively conservative. Permutation testing [4] provides a means of controlling family-wise error rates without being overly conservative, but it is often considered to be infeasible due to computational constraints. Beagle is a new program that performs single-marker and multilocus analysis, and adjusts for the multiple tests performed with permutation testing, while being computationally fast even on large data sets [3]. The type of multilocus test that Beagle uses is a localized haplotype cluster test [5].

Genetic Analysis Workshop (GAW) 15 Problem 2 involved studies designed to investigate genetic risk factors for rheumatoid arthritis. One of these studies involved a dense panel of 2300 single-nucleotide polymorphisms (SNPs) that were genotyped in cases and controls on a 10 megabase region of chromosome 18q. This region has shown evidence for linkage to rheumatoid arthritis in U.S. and French linkage scans [6,7]. The genotyped individuals included 460 rheumatoid arthritis cases and 460 controls collected by the North America Rheumatoid Arthritis Consortium (NARAC). The controls were recruited from a New York City population [GAW15 Problem 2 data description].

We applied a localized haplotype-cluster test [5] to these data using Beagle, and found an association with a rare protective haplotype located upstream of the *CCBE1 *gene. Other types of multilocus test confirmed this association.

## Methods

### Quality control and ethnicity

As a quality control measure, we tested for Hardy-Weinberg disequilibrium (HWD) in controls using an exact test. One marker had a HWD *p*-value of 3.1 × 10^-12 ^(SNP194), which is clearly significant after correction for multiple testing. We removed all markers with *p*-values less than 10^-4^, which was a total of five markers (SNPs 194, 386, 794, 811, 1957). We also checked rates of missing data. One case individual (Family ID 09082 and individual ID 200) had no genotypes, and was removed from the analysis. All other individuals had at most 6% missing data, and all SNPs had at most 8% missing data, which we felt was acceptable.

All the individuals self-identified as White. All but 144 also provided one or more ethnic subclassifications. These are shown in Table 1. To try to keep the sample ethnically homogenous without reducing the sample size too much, we excluded any individuals with ethnicities shown in the right column of Table 1. In total, 74 individuals were excluded on these grounds. This ethnicity exclusion was performed prior to analysis. Subsequent to the association analysis, we further investigated the ethnicity of individuals carrying a significant haplotype that was identified in the association analysis.

**Table 1:** Numbers of individuals reporting the ethnicities shown

Ethnicities included		Ethnicities excluded	
Northern European	536	East Mediteranean	16
Scandinavian	119	Ashkenazi Jew	3
Southern European	55	Middle East	2
Central European	267	American Indian/Alaska Native	49
Eastern European	75	American Indian/North America	13
French Canada	13	American Indian/South & Central America	11
South Africa	2	Latino/Hispanic	4
		Latino/Mexican	2
		Latino/Puerto Rican	2
		Native Hawaiian/Pacific Islander	1
		Native Hawaiian/Hawaiian	1

### Localized haplotype cluster analysis

The localized haplotype cluster method [5] empirically models the linkage disequilibrium structure in densely spaced genetic markers to derive haplotype clusters that are localized to specific positions. The approach is similar to a haplotype block approach, in that it adapts to local linkage disequilibrium, but it is more flexible and does not impose a block structure on the data. The fitted haplotype clusters are tested for association with case-control status, and permutation of case-control status can be used to correct for multiple testing [3,4].

The localized haplotype cluster method takes as input phased haplotype data with imputed missing values. We used fastPHASE version 1.1 [8] to do the phasing and imputation. We found that with this amount of data, increasing the number of clusters (parameter K) improved the quality of the phasing (measured by accuracy in inferring masked data), as did increasing the number of iterations of the expectation-maximization (EM) algorithm (parameter C). We used C = 40 and K = 30 for the haplotype data used in subsequent analyses, although using default values (C = 25 and K = 10) gave almost identical results in the localized haplotype cluster analysis. Haplotypes were phased without regard to trait status.

Using the phased haplotypes, we fit a localized haplotype model. We used the fitted model to perform localized haplotype cluster tests [5] (4345 tests; Fisher's exact test applied to each haplotype cluster). We also performed single-marker allelic tests, with inferred missing data from the fastPHASE analysis, using Fisher's exact test (2295 tests). Using permutation, we adjusted for multiple testing, adjusting simultaneously for both classes of test (a total of 6640 tests). All these analyses were conducted with the Beagle program [3].

### Other analyses

For comparison with the localized haplotype cluster tests, we ran a multilocus score test [9] and a haplotype block-based test using Haploview version 3.32 [10]. We also used Haploview to investigate the linkage disequilibrium structure around an area showing significant association. Default settings were used for Haploview except where otherwise noted.

## Results

### Significant haplotype association with rheumatoid arthritis

The localized haplotype cluster analysis detected an association that was significant after adjustment for multiple testing. The *p*-value for this cluster was 6.13 × 10^-6 ^before adjustment for multiple-testing, and 0.012 after adjusting for multiple testing (10,000 permutations), including adjustment for single-marker tests as well as all the localized haplotype cluster tests [3]. None of the single-marker test *p*-values were less than 0.2 after adjusting for multiple testing.

The significant haplotype cluster consisted of haplotypes having the sequence 2,1 at SNPs 1631 (rs2195534) and 1632 (rs1791320). These SNPs are located less than 500 base pairs upstream of *CCBE1 *(collagen and calcium binding EGF domains 1) on chromosome 18q21.32 (NCBI build 36.2, dbSNP build 126). SNPs 1515 to 1630 are located within this gene. Haplotypes in the sample with this 2,1 sequence at SNPs 1631 and 1632 also all share the sequence 1,1,2,1,1 at SNPs 1626 to 1630. A total of 32 of the individuals included in the analysis have this haplotype, of whom 29 are controls, so the haplotype is associated with reduced risk of rheumatoid arthritis. There was also one carrier (a case individual) among the individuals excluded from the analysis on grounds of ethnicity.

**Figure 1:**
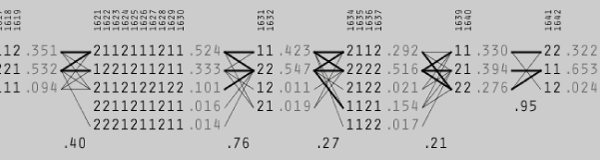
**Block structure around significant haplotype**. The significant haplotype is "21" in the block containing SNPs 1631 and 1632. Thick lines represent proportions >10%, while thin lines represent proportions >0.5%.

**Figure 2:**
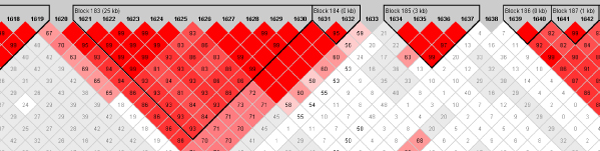
**LD structure around SNPs 1631 and 1632**. D' values are shown.

Figures 1 and 2 from Haploview show the linkage disequilibrium structure around the significant haplotype. SNPs 1631 and 1632 form a haplotype block that is in fairly strong LD with a block comprising SNPs 1621–1630 (Fig. 2).

Eight of the 32 carriers included in the analysis have some Eastern European ancestry, whereas only 72 of the 845 individuals included in the analysis have Eastern European ancestry, so the haplotype is enriched in this ethnic group. All other ethnic groups had similar frequencies in the overall sample and in the carriers. All 8 individuals with Eastern European ancestry carrying the haplotype were controls. The 72 individuals with Eastern European ancestry included 37 cases and 35 controls. If the individuals with Eastern European ancestry are removed from the analysis, the haplotype is no longer significant after adjustment for multiple testing.

### Comparison with other analysis methods

Single-marker allelic tests did not detect significant associations after correction for multiple testing. The unadjusted *p*-values for single marker tests of SNPs 1631 and 1632 were 1.0 and 0.2, respectively. The smallest unadjusted *p*-value in the region consisting of SNPs 1620–1632 was 0.01 for SNP 1621, however the multiple-testing adjusted permutation *p*-values for all markers in this region were 1.0.

We ran a multilocus score test [9] with SNPs 1631 and 1632 on the same individuals used in the localized haplotype clustering tests. For this test, the genotypes at the SNPs are coded as 0, 1, or 2 depending on the number of copies of one of the alleles. Individuals with a missing genotype at one of the two SNPs were removed from the analysis (four individuals). The test statistic was 15.0, yielding a *p*-value of 5.6 × 10^-4 ^(tail of chi-square distribution with 2 degrees of freedom). We did not attempt to adjust for multiple testing because it is unclear what set of tests should be considered, but we note that the score test *p*-value is almost 100 times higher than that obtained using localized haplotype clustering (6.1 × 10^-6^) and thus would probably not survive correction for multiple testing of all relevant marker sets in the data.

We used Haploview [10] to test for association between haplotypes defined by blocks (default settings) and case-control status, using the raw genotype data, with the same individuals used as in the other analyses. In total, 2300 single-marker tests and 1135 haplotype tests (from 264 blocks) were performed. The minimum unadjusted *p*-value was 1.7 × 10^-5 ^obtained for haplotype "21" in the block made up of SNPs 1631 and 1632. In 1000 permutations, 24 obtained minimum *p*-values lower than the original minimum *p*-value, so that the multiple-testing adjusted *p*-value is 0.024, which is higher than that found using the localized haplotype cluster method. Although Haploview did not require data to be phased before input, it did take longer to do the permutation testing, with 1000 permutations running overnight, compared to 4 minutes for the 10,000 permutations of the haplotype cluster test with Beagle. Whereas Haploview takes a full likelihood-based EM approach to haplotype testing, Beagle uses inferred haplotypes, and constructs haplotype clusters without regard to case-control status, so that permutation-testing does not need to redefine the clusters or re-estimate the haplotypes and is very fast (for details, see Browning and Browning [3]).

## Conclusion

Large genetic data sets such as this one pose computational and statistical challenges. By using the Beagle software [3], we were able to use permutation to adjust for the multiple tests performed, including both single-marker and localized haplotype cluster tests. We detected a significant association with a protective haplotype located upstream of the *CCBE1 *gene. This haplotype is over-represented in Eastern European individuals relative to other ethnicities in the sample, but the association does not appear to be a population structure artifact as roughly equal numbers of Eastern European cases and controls were included in the sample.

## Competing interests

The author(s) declare that they have no competing interests.
